# Stem Cell-Derived Hepatocyte-Like Cells as Model for Viral Hepatitis Research

**DOI:** 10.1155/2019/9605252

**Published:** 2019-06-12

**Authors:** Jingjing Wang, Bingqian Qu, Fang Zhang, Cindy Zhang, Wanyan Deng, Viet Loan Dao Thi, Yuchen Xia

**Affiliations:** ^1^State Key Laboratory of Virology, School of Basic Medical Sciences, Wuhan University, Wuhan, China; ^2^Department of Infectious Diseases, Molecular Virology, University Hospital Heidelberg, Heidelberg, Germany; ^3^Department of Translational Medicine, Baruch S. Blumberg Institute, Doylestown, PA, USA; ^4^Schaller Research Group at Department of Infectious Diseases, Molecular Virology, Heidelberg University Hospital, Cluster of Excellence CellNetworks, Heidelberg, Germany; ^5^BioQuant Center, University of Heidelberg, Heidelberg, Germany; ^6^German Center for Infection Research (DZIF), Heidelberg, Germany; ^7^Key Laboratory of Molecular Biology for Infectious Diseases (Ministry of Education), Institute for Viral Hepatitis, Department of Infectious Diseases, The Second Affiliated Hospital, Chongqing Medical University, Chongqing, China

## Abstract

Viral hepatitis, the leading cause of liver diseases worldwide, is induced upon infection with hepatotropic viruses, including hepatitis A, B, C, D, and E virus. Due to their obligate intracellular lifestyles, culture systems for efficient viral replication are vital. Although basic and translational research on viral hepatitis has been performed for many years, conventional hepatocellular culture systems are not optimal. These studies have greatly benefited from recent efforts on improving cell culture models for virus replication and infection studies. Here we summarize the use of human stem cell-derived hepatocyte-like cells for hepatotropic virus infection studies, including the dissection of virus-host interactions and virus-induced pathogenesis as well as the identification and validation of novel antiviral agents.

## 1. Introduction

Viral hepatitis manifests itself as continuous liver inflammation and eventually liver injury and hepatic failure. As summarized in Table 1, the major causative agents of viral hepatitis are five hepatotropic viruses, including hepatitis A virus (HAV), hepatitis B virus (HBV), hepatitis C virus (HCV), hepatitis D virus (HDV), and hepatitis E virus (HEV). HAV and HEV normally spread through contact with contaminated water or food, resulting in an estimated annual incidence of 1.5 million HAV infections and 20 million HEV infections [1, 2]. Both HAV and HEV typically cause acute infections; however, HEV can also cause chronic infections in immunocompromised patients [2]. HBV, HCV, and HDV are transmitted through blood transfusions, organ transplants, sex, and injection behavior [3–5]. Approximately, 10–15% of chronically HBV-infected patients are coinfected with HCV and 5% with HDV [6]. Infection with HBV, HCV, and HDV can cause both self-limited and chronic hepatitis and is the leading cause of liver diseases including fibrosis, cirrhosis, and hepatocellular carcinoma (HCC) [3, 4, 7]. In order to prevent disease progression, early diagnosis and treatments are vital. In spite of recent extraordinary advances in the treatment of hepatitis C, based on the success of HCV basic research [8], the need remains to understand the underlying molecular and cellular mechanisms of liver pathogenesis caused by the other hepatotropic viruses. The development of novel specific drugs against hepatotropic virus infection has been a challenging task, partially due to the lack of physiologically relevant cell culture models that can be used for medium/high-throughput drug screening.

Hepatoma cells have been invaluable across the history of hepatotropic virus studies in cell culture. Yet, their aberrant intracellular signaling and metabolic activities limit investigations of the viral and cellular innate immunity interactions as well as effects on cellular proliferation, metabolism, and apoptosis pathways. Furthermore, most hepatoma cell lines lack various functional enzymes, such as CYP450 and other phase I, II, and III drug-metabolizing enzymes, which make them unsuited for the assessment of antiviral drug interactions and metabolism [9, 10]. Therefore, studies using these models are limited in their ability to mimic natural virus-induced pathologies in the liver.

The most authentic cell culture system for hepatotropic virus studies is primary human hepatocytes (PHH). Yet, their use is hindered by limited donor supply, donor-to-donor variability, and rapid dedifferentiation upon plating in cell culture [11]. Given the limitations and challenges of using hepatoma cells and PHHs, hepatocyte-like cells (HLCs) derived from human embryonic stem cells (hESCs) or induced pluripotent stem cell (iPSC) have emerged as a promising cell culture model to study basic and translational liver diseases as well as hepatitis virus infection [12–14]. HLCs have been differentiated from diverse resources, such as hESCs, iPSCs, liver-resident hepatic progenitor cells, and bone marrow-derived mesenchymal stem cells [15]. Differentiated HLCs are functionally characterized by the production of urea, indocyanine green uptake, glycogen storage, and inducible cytochrome P450 (CYP450) activity [16]. In addition, they can rescue liver function after transplantation into animal models [17]. To date, HLC infection models for HBV, HCV, and HEV have been successfully established [17–19]. Here, we summarize the current knowledge on cell culture-based models available for these viruses and highlight the advantages of HLCs derived from stem cell as an improved system for basic and translational viral hepatitis research.

## 2. Hepatocyte-Like Cells for HBV Infection

Despite the availability of an efficient prophylactic vaccine, HBV infection is still a global public health burden with an estimated 257 million chronically infected people who are at increased risk of developing liver related-fibrosis, cirrhosis, and hepatocellular carcinoma (HCC) [25]. HBV contains a partially double-stranded, relaxed circular DNA (rcDNA) genome of approximately 3.2 kb, covalently linked to the HBV polymerase [26]. The rcDNA is delivered into the nucleus after viral entry and converted into fully double-stranded DNA, which is itself converted by ligation into an intracellular HBV replication intermediate called covalently closed circular DNA (cccDNA). cccDNA is responsible for HBV persistence in infected cells [6, 26]. A curative treatment of chronic hepatitis B should therefore target permanent transcriptional silencing or elimination of cccDNA [27].

Currently, treatments for chronic hepatitis B are limited to type 1 interferons (IFN-*α*) and five approved nucleos(t)ide analogues (NAs) [28]. Due to severe side effects of interferon therapy, only few patients are eligible for treatment, and less than 10% of them show a sustained virological response evidenced as loss of hepatitis B surface antigen (HBsAg) [29]. NAs are the most potent drugs; tenofovir and entecavir can reduce viral DNA, often below the detection limit with low resistance development [30, 31]. However, most patients remain HBsAg-positive even after prolonged treatment and the frequent viral rebound upon therapy withdrawal indicates a need for lifelong treatment [32]. In addition, long-term administration of tenofovir has been associated with Fanconi syndrome, a decrease in bone mineral density and chronic renal tubular damage [33]. Since current antiviral strategies cannot completely eradicate viral infection, an urgent need for the development of novel antiviral therapeutics remains [34].

Basic and translational research has been hindered by the absence of *in vitro* experimental models that feature the physiological condition of hepatocytes and permits efficient HBV and infection. As shown in Table 2, human hepatoma cell lines, such as Huh-7 and HepG2, are widely used as surrogate models for HBV infection, even though they only partially mimic physiological hepatic functions. Stable HBV-integrated hepatoma cell lines have been generated through transfection of human hepatoma cells with an HBV-expressing plasmid [35–38]. Alternative systems were the delivery of the HBV genome by baculoviral or adenoviral vectors, which resulted in sufficient HBV replication and viral particle production [39, 40]. However, these cell lines are not permissive for natural infection as they are unable to mediate early steps of virus infection, including entry, uncoating, and cccDNA formation. Primary human hepatocytes (PHHs) support the full viral replication cycle and serve as the gold standard of HBV infection. However, they have many disadvantages, including high donor variability, short lifespans, and limited availability. Despite many attempts to improve methods for maintaining freshly isolated PHHs, they often rapidly dedifferentiate in culture dishes [19, 41–43]. HepaRG cells are liver progenitor cells that can be differentiated in vitro and then support the whole HBV life cycle, an alternative tool for HBV studies [35, 44]. However, the efficiency of HBV infection in these differentiated HepaRG cells remains lower than in other cell systems. In addition, the differentiated cells contain both hepatocyte and biliary lineages, which affects HBV-host interaction studies in a hepatocyte-specific environment [45]. New HBV infection cell culture models have been developed when human sodium taurocholate cotransporting polypeptide (NTCP) was identified as the HBV entry receptor [46]. NTCP-overexpressing hepatoma cell lines were generated including HepG2-NTCP and Huh-7-NTCP cell lines, which provide an easily accessible platform for HBV-host interaction and antiviral studies [46, 47]. But, as mentioned above, although the entire HBV life cycle is recapitulated, hepatoma cells have altered physiological signaling pathways.

**Figure 1:**
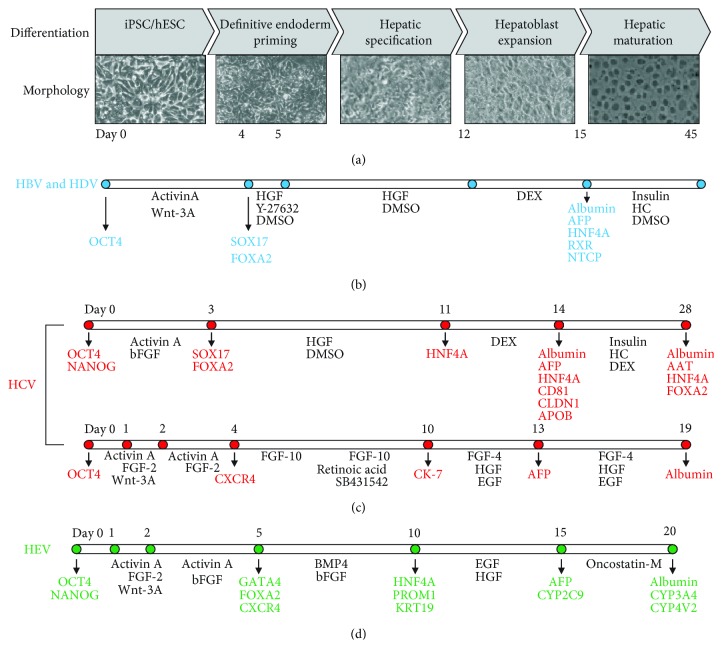
Stem cell-derived hepatocyte-like cells support virus research of HBV, HCV, and HEV. (a) During differentiation, iPSC or hESC undergo definitive endoderm induction, hepatic specification, hepatoblast expansion, and hepatic maturation to become HLCs that are permissive for infection with hepatitis viruses. (b) For HBV infection, cells are treated with activin A and Wnt-3A enhancer for 4 days and HGF and Rock inhibitor Y-27632 for one day, followed by HGF for 1 week. Hepatoblast cells are administered with DEX for 3 days and can be used for HBV infection at this point. The infected cells can be maintained in the presence of insulin, HC, and DMSO for another 1 month [19]. (c) For HCV application, iPSC/hESC is differentiated using two methods. Upper: this arm is similar to (b) (a, [17]). Lower: cells are treated with activin A and FGF-2 until day 4 when FGF-10 is added for 2 days and then coadded with retinoic acid and TGF-*β* inhibitor SB431542 until day 10. Cells are further treated with FGF-4, HGF, and EGF for about 10 days [65]. (d) On day 5, endoderm is formed by activin A, FGF-2, and bFGF and further differentiated by BMP4 and bFGF until day 10. Cells are terminally differentiated by EGF and HGF to hepatocyte-like cells. Cells can be infected with HEV or maintained in the medium containing oncostatin-M [18, 75]. Abbreviations: AAT: *α*-1-antitrypsin; AFP: alpha fetoprotein; APOB: apolipoprotein B; BMP4: bone morphogenetic protein 4; Cd81: cluster of differentiation 81; CK-7: cytokeratin 7; CLDN1: claudin-1; CXCR4: C-X-C chemokine receptor type 4; CYP: cytochrome 450 enzyme; DEX: dexamethasone; DMSO: dimethyl sulfoxide; EGF: epidermal growth factor; FGF: fibroblast growth factor; FOXA2: forkhead box protein A2 (also known as hepatocyte nuclear factor 3-beta); GATA4: GATA-binding protein 4; HC: hydrocortisone; hESC: human embryonic stem cells; HGF: hepatocyte growth factor; HNF: hepatocyte nuclear factor; iPSC: induced pluripotent stem cells; KRT19: cytokeratin 19; NANOG: Nanog homeobox; NTCP: sodium taurocholate cotransporting polypeptide; OCT4: octamer-binding transcription factor 4; PROM1: prominin 1; RXR: retinoid X receptor; SOX17: sex determining region Y-box 17; TGF: tumor growth factor.

Recently, iPSC-derived HLCs were reported to support HBV infection [19, 63]. A time-course experiment showed that both a full activation of the transcription machinery and an expression of NTCP on the cell surface are essential to achieving productive HBV infection. This demonstrated the potential of human iPSC-derived HLCs for *in vitro* studies of HBV biology, yet the infection efficiency remained very low. Although the authors observed temporal induction of interferon-stimulated genes (ISGs) in HBV-infected HLCs, studies from other groups rather support the notion that HBV is a stealth virus both *in vitro* and *in vivo* [68–71]. Similarly, Sakurai et al. established human iPSC-derived HLCs that allow about 20% HBV infection efficiency [62]. Xia et al. used an optimized protocol [72] to differentiate the non-colony-type monolayer culture of hESCs and iPSCs to HLCs in 15 days (Figure 1). The HLCs maintained their differentiated state and allowed HBV infection for more than 4 weeks. Importantly, the authors successfully demonstrated that the optimized protocol for HLC differentiation provided an *in vitro* model capable of supporting HBV spread. Notably, the dedifferentiation process occurred at a slower rate in HLCs than in PHHs, as high expression levels of proviral factors, including NTCP, HNF4A, and RXRA, were maintained for more than 3 weeks, making them a suitable model for long-term HBV infection studies [19]. Knocking down NTCP reduced HBV infection while knocking down antiviral factor APOBEC3A enhanced viral replication, indicating that HLCs constitute an appropriate system for virus-host interaction studies. By using this model, the authors identified two host-targeting agents, genistin and PA452, as novel antivirals. Recently, Nie et al. used iPSCs to generate liver organoids and evaluated their application in studying HBV virus–host interactions [73]. They cultured iPSC-derived endodermal, mesenchymal, and endothelial cells with a chemically defined medium in a three-dimensional (3D) microwell culture system, in which the cells organized themselves to gradually differentiate into a functional liver organoid. They showed that the organoid exhibited stronger hepatic functions than did 2-cultured HLCs with a higher susceptibility to HBV infection [73]. Yuan et al. developed a mouse model to study *in vivo* HBV infection by engrafting iPSC-derived HLCs into immune-deficient mice [74]. The liver of these mice contains approximately 40% HLCs at week 6 and maintained at this level for at least 14 weeks. After HBV infection, viral replication markers such as HBsAg, HBeAg, RNA, DNA, and cccDNA were detectable in the sera. Furthermore, these mice can be used to test different antivirals [74]. Together, all these studies demonstrated that HLCs fully support HBV infection and virus-host interactions, allowing the identification and validation of novel antiviral agents.

## 3. Hepatocyte-Like Cells for HCV Infection

Around 71 million people worldwide are chronically infected with HCV, which increases their risk of progressive liver disease [76]. Although the standard of care for chronic HCV infection has been dramatically improved through direct-acting antiviral agents (DAAs), it still poses significant problems, including treatment failure in some patient groups and limited access to therapy due to high cost of treatment. Further, a protective vaccine is still in need. Notably, 15–45% of HCV-infected individuals are able to clear the virus within six months without intervention, but the underlying mechanisms remain unknown [77, 78].

To address these scientific questions and develop new anti-HCV drugs, various *in vitro* HCV-infection models have been developed. JFH-1 is a unique cell culture- (cc-) adapted strain of HCV to study the complete viral life cycle in the hepatoma system [79]; however, it does not reflect the variability and diversity of HCV infection in patients [80]. The Huh-7.5.1 cell line derived from Huh-7 cells that carries a defect retinoic-inducible gene I (RIG-I), a critical player in viral genome recognition and host immune response, is frequently used for JFH-1 HCVcc infection [81].

Several studies have independently validated that HLCs are competent to support HCV infection (Table 2). In preliminary research, HLCs were generated from iPSCs differentiated with growth factors and by adenovirus delivery of SOX17, HEX, and HNF4A. All HCV entry receptors were expressed on these HLCs, including CD81, SR-B1, claudin-1, and occludin, which allowed the entry and replication of HCV pseudoparticles and subgenomic replicons, respectively [82]. Subsequently, other groups demonstrated that HLCs are not only permissive to different forms of cell culture-adapted HCV (viral pseudoparticles and JFH-1; HCVpp and HCVcc) but also showed detectable infection with different serum-derived HCV genotypes 1a, 1b, 2, 3, and 4; this is not possible with hepatoma cell lines since they are not permissive for infection with patient isolates [17, 83].

HLCs support the complete life cycle of HCV genotype 2a for up to 21 days (Figure 1) [65, 68]. Another feature of HLCs is that HCV can spread from infected cells to adjacent cells, suggesting possible direct cell-to-cell transmission of HCV, as has been described previously in HuH-7 cells [17]. A recent study used human iPSCs derived from human mesenchymal stem cells that were subsequently differentiated into HLCs with polycistronic OSKM-reprogramming factors. These HLCs supported the entire life cycle of wild-type HCV (genotype 1a, 1b, 3a, 3b, 6f, and 6n) isolated from patients and achieved increasing infection rates by incubating cells with *α*-tocopherol. The released HCV viral particles could infect both naïve HLCs and HuH-7 cells and were susceptible to treatment with IFN-*α*, ribavirin, or sofosbuvir [85].

Although HLCs represent a unique and highly relevant model to study HCV infection *in vitro* and *in vivo*, particularly in the context of a patient-specific genetic background, some limitations of this model remain to be addressed. First, the production of viral particles remains very low compared with reported levels from HuH-7 cell lines [86]. It has been shown that the permissive and persistent infection of HCV in hepatic progenitor cells is affected by liver-specific microRNA-122 and cellular cytokines [87, 88]. By inhibiting the JAK/STAT pathway to block IFN responses, viral infection and replication were improved in HLCs [89]. Additionally, higher HCV replication levels were observed in STAT2- but not STAT1-deficient HLCs [89]. Even after JAK/STAT pathway inhibitor treatment, HLCs demonstrated intact type III interferon and ISG responses. This suggests that HLCs may be a suitable model to study the HCV-host interaction [86]. Multiple mutations in different regions of the viral genome of JFH-1 HCVcc enhanced the titers in HuH-7 cells, and infections were maintained in an animal model, but the appearance of mutations has not been investigated in the HLCs. A further concern in the HLC model is the observed variability between cell lines with timing or cytokine concentrations necessary for hepatocyte differentiation. Thus, iPSC differentiation protocols and HLC culture conditions need to be optimized. For example, humanized liver chimeric mice based on human hepatocyte (such as HLCs) engraftment were reported to support HCV infection [90]. Engrafting HLCs *in vivo* to produce human liver chimeric mouse models has been fraught with low efficiencies [91]. By using an optimized hepatocyte differentiation protocol on transgenic mice, which carry the uPA (urokinase-type plasminogen activator) gene driven by the major urinary protein promoter onto a SCID (severe combined immunodeficiency)/beige background, HLCs differentiated from both hESCs and patient-specific iPSCs were able to engraft and undergo further maturation *in vivo*. Productive and chronic HCV infection in these repopulated liver injury models can be launched with high-dose inoculations (1,000 CID50 per mouse) [17]. Although challenges remain, robust cell culture and animal models for serum-derived HCV using HLCs provide remarkable systems for investigating HCV life cycle and HCV-associated hepatocellular carcinoma development.

## 4. Hepatocyte-Like Cells for HEV Infection

HEV is recognized as an important global health problem [92]. HEV is a nonenveloped positive-strand RNA virus of the *Hepeviridae* family, which is divided into two genera: *Orthohepevirus* and *Piscihepevirus* [93]. The *Orthohepevirus* genus is further divided into four species A, B, C, and D. Human-infecting HEV strains belong to the *Orthohepevirus* A, which include human-restricted genotypes (gt) 1 and 2 as well as zoonotic genotypes 3, 4, and 7. The human-restricted genotypes are transmitted fecal-orally and sporadically lead to large waterborne outbreaks in developing countries with poor sanitation (reviewed in [94]). These infections are mostly acute and self-resolving but can cause an increased virulence in pregnant women, leading to a 25% maternal mortality in the third trimester [95]. For the zoonotic viruses, infected animals serve as reservoirs and can transmit HEV through the consumption of infected meat [94]. These zoonotic species of HEV cause acute and chronic diseases in immunocompromised patients [92]. Reducing immunosuppression in combination with using off-label ribavirin is the only available treatment, but treatment resistances have been reported [96–98]. A high-efficacy vaccine has been developed and licensed in China but is not available elsewhere [24].

The 7.2 kb polyadenylated HEV genome contains three partially overlapping open reading frames (ORF1-3) (reviewed in [2]). ORF1 encodes the viral replicase, ORF2 for the capsid, and ORF3 for a small protein involved in virus assembly and secretion [2]. A range of different expression systems have been used to study HEV without resulting in authentic virus replication [67]. In this regard, HEV behaves like other hepatotropic viruses, in that they grow poorly in cell culture, which has severely hampered molecular studies, leaving many fundamental aspects of its life cycle poorly understood [67].

Breakthroughs in developing robust HEV cell culture systems have been made through the isolation of specific viral strains with improved replication efficiency and the identification of compatible cell lines [99]. After serial passaging in these cell lines, the isolated strains accumulated mutations and/or insertions, which increased their ability to replicate. For example, a gt3 HEV virus, the Kernow-C1 strain, was isolated from a chronic HEV patient [100] and serially passaged six times (passage 6) in the hepatoma cell line HepG2 [101]. A virus with an insertion derived from the human 40S ribosomal protein S17 in the ORF1 region became the dominant species with greater *in vitro* replication ability and broadened host range [101]. Similarly, other strains with insertions into ORF1 have been reported with enhanced viral fitness *in vitro* [102–104]. These adapted clones enabled molecular HEV studies and yielded valuable insights into HEV biology. Yet, this approach is limited to gt3 and 4 viruses and, to our knowledge, has not been successful for other genotypes.

Studies have proposed utilizing HLCs differentiated from iPSC/hESC as an alternative HEV cell culture model to hepatoma cells and PHHs (Figure 1) [18, 66]. HLCs can be infected with the adapted p6 strain [18, 66, 75]. Transition studies showed that germ layer cells support intracellular HEV replication but not infection [66]. Only when endodermal cells were differentiated to immature hepatocytes did they become susceptible for HEV infection [18]. This strongly suggested that virus entry, governed by the expression of a yet unknown cellular protein, was the limiting factor which could also be the key determinant of HEV tissue tropism [66]. In addition, HLCs are readily permissive for HEV isolates from animals infected with gt1-4 without prior adaptation [18]. Surprisingly, an early, nonadapted passage of the Kernow-C1 strain replicated better than the adapted p6 strain in HLCs. This suggests that acquired mutations in cell culture attenuate viral replication in more physiologically relevant systems. HLCs therefore enable studies of not only authentic HEV replication but also pan-genotype HEV biology. The high degree of heterogeneity among HEV genotypes has not been fully explored to date, but HLCs may now provide a reproducible platform to study such differences. As such, viral or cellular determinants that may define a host range and infections across species barriers have not been defined yet and are one of the many poorly understood topics in the field.

Employing precise editing technologies, such as CRISPR (cluster regularly interspaced short palindromic repeats)/Cas9 (CRISPR-associated protein 9), allows for rapid and efficient genome-editing of relevant host factors in stem cells to explore their importance in HEV replication pathways. Using CRISPR-Cas9, we identified at least one striking difference between nonadapted and adapted HEV replication [18]. The host factor cyclophilin A, which was previously reported to restrict HEV replication [18], only inhibited the cell culture-adapted p6 clone but not the original Kernow-C1 isolate replication in HLCs [18]. Corroborating evidence has shown discrepancies in drug responsiveness between *in vitro* and *in vivo* conditions; molecules (i.e., mycophenolic acid and rapamycin) that affected adapted HEV replication in cell culture [105, 106] failed to show any effect in patients [107]. If this discrepancy is due to alterations of the viral genome or a reflection of *in vivo* complexity as opposed to viral replication studies in a single cell type, it can be now explored using HLCs. With recent efforts in identifying novel compounds that inhibit HEV replication [75, 108–110], validation in the HLCs system will become more and more relevant.

Several studies suggest that host genetics determine susceptibility to HEV infection [111–113]. The ability to study replication of nonadapted HEV isolates in tandem with autologous, patient-derived iPSCs enables personalized models of HEV infection [67]. This may provide patient-tailored platforms to test potential treatments *in vitro*, especially for chronic HEV patients who have already developed resistance against RBV. An alternative treatment approach that we are currently exploring is based on the use of nonpathogenic adeno-associated viruses (AAV) combined with CRISPR-Cas9 to deliver short hairpin RNA (shRNA) to downregulate HEV replication. In this scenario, genetic vaccination would be achieved by transducing patient-derived iPSCs prior to HLC differentiation and transplantation into the liver of chronic HEV patients to establish a genetically protected hepatocyte population. Alternatively, hepatotropic AAVs will allow direct delivery of target shRNAs *in vivo*. These approaches are not restricted to HEV, as HLCs are permissive for HCV and HBV isolates [63, 65] (Table 1). With that, HLCs provide a uniquely reproducible and genetically tractable cellular system in which to perform coinfection studies as widely used hepatoma cell lines vary in their virus permissiveness (Table 2).

Beyond HLCs, stem cell technology may also provide a platform to study other aspects of HEV biology. HEV mainly infects the liver but likely additional tissues [114] as some patients experience extrahepatic manifestations including neurological disorders, thrombocytopenia, renal injury, and other conditions [115]. This is further corroborated by the observation that HEV can replicate *in vitro* in nonhepatic cell types, such as lung [116], neuronal [117], and placental [118] cell lines. Stem cells, with their intrinsic ability to give rise to cells of various lineages, may help to define the determinants of HEV tissue tropism [66]. In conclusion, knowledge on the viral life cycle of HEV and virus-host interactions, i.e., on systemic and cellular levels, remains scarce. Studies of pan-genotype HEV biology in a physiologically relevant cell system such as HLCs, which support authentic HEV infection and replication, shall significantly advance our understanding of HEV biology. This will facilitate and promote the development of specific anti-HEV therapies.

## 5. Conclusions and Future Directions

HLCs derived from hiPSC or hESC provide a promising tool to study the biology of hepatotropic viruses and to screen novel antiviral treatments in the future. We summarized current progress in developing HLCs that support the entire life cycles of HBV, HCV, and HEV (Figure 1). HLCs constitute a novel cell culture model that is more physiologically relevant than immortalized hepatoma cell lines. Beyond that, the use of HLCs may help overcome two major limitations of PHHs: donor-to-donor variability and long-term culture to study chronic infection. Specifically, HLCs support high-efficiency and long-term HBV replication and, remarkably, virus spread [19]. In terms of the nature of diverse genotypes and high replicative mutations of RNA viruses, such as HCV and HEV, HLCs allow pan-genotype permissiveness and even support direct infection with patient-derived isolates that have not been adapted in cell culture. Engrafting HLCs into immunosuppressive liver injury mouse models, like the uPA/SCID mice, may facilitate studies of antiviral evaluation and virus-host interaction *in vivo*.

Coinfections of hepatitis viruses (e.g., HBV, HCV, and HEV) occur in patients. However, the exact modes of coinfection are poorly described due to the single permissiveness of available culture models (Table 2). HLCs therefore constitute a universal tool, in which to study how two or more hepatitis viruses modulate host factor(s) such as MAVS and cyclophilin. This may provide information on how and in which order coinfected patients could be treated.

HLCs derived from iPSCs are of less societal and ethical concern than PHHs or fetal tissue-derived hepatocytes are. In addition, HLCs serve as a powerful tool to assess the influence of genetic factors on virus infection, as HLCs can be generated from iPSCs with a diverse genetic background.

Despite these advantages and optimization of available protocols, HLC differentiation remains time-consuming and complicated. Although HLCs are more physiologically relevant than many hepatoma cell lines, they retain an immature phenotype and cannot fully recapitulate hepatocyte functions. Perhaps, differentiation under 3D-culture conditions may improve this and yield HLCs that resemble PHHs more closely. Supporting this, Gieseck et al. have demonstrated that hepatocyte-specific genes are higher expressed in HLCs, when cultured in 3D conditions [119].

Ultimately, HLCs provide a personalized platform for viral hepatitis studies. For patients who do not respond to available treatments, personalized iPSC-derived HLCs are the best model to study the host determinants and validate second-line antivirals. Taken together, HLCs provide an important tool for studying the life cycle of hepatitis viruses, in spite of the distinct replicative nature of HBV, HCV, and HEV. The development of HLCs derived from stem cells has opened a new era and provides a physiologically relevant system to advance our understanding of the viral life cycles. This will ultimately contribute to the development of novel therapeutic strategies towards the elimination of viral hepatitis.

## Figures and Tables

**Table 1:** Overview of hepatitis viruses.

	HAV	HBV	HCV	HDV	HEV
Classification	Picornavirus	Hepadnavirus	Hepacivirus	Deltavirus	Hepevirus

Genome	+ssRNA	dsRNA-RT	+ssRNA	-ssRNA	+ssRNA

Incubation (days)	20-40	45-160	15-150	30-60	15-60

Transmission	Fecal-oral	Parenteral Perinatal Sexual	Parenteral Perinatal Sexual	Parenteral Sexual	Fecal-oral

Chronicity	Acute	5-10% chronic^1^ 80% neonates	70% chronic	Coexistence with HBV	Acute^2^ (Chronic in immunocompromised patients)

Natural host	Human Chimpanzee Monkey [20]	Human^3^ [21]	Human^3^ [22]	Human^3^ [23]	Human Animal^4^ [24]

Carcinogenesis	–	+	+	–	–

Prophylaxis	Vaccine	Vaccine	NA	HBV vaccine	Vaccine^5^

Therapy	NA	IFN, NAs	DAAs	IFN	RBV and withdrawal of immunosuppressants

Cure	Self-cure	No	Yes	No	Self-cure (yes)

^1^5-10% in immunocompetent adults; ^2^mild in normal patients and severe in pregnant women; ^3^chimpanzees are susceptible but not naturally infected; ^4^genotype 3- and 4-specific; ^5^licensed in China. ss: single-stranded; ds: double-stranded; RT: reverse-transcriptase; IFN: interferon-*α*; NAs: nucleos(t)ide analogues; DAA: direct-acting antiviral; RBV: ribavirin; NA: not applicable.

**Table 2:** Receptors and infection models of hepatitis viruses.

	HAV	HBV	HCV	HEV
Receptor	TIM-1 [48]	NTCP [46]	CD81, SR-BI, OCLN, CLDN1 [49–52]	Unknown
Cell model	HAV	HBV	HCV	HEV
PHH	+[53]	+[46]	+[54]	+[55]
HepG2	+[53]	+/–	–	+[56]
HepG2^NTCP^	Unknown	+[46]	–	Unknown
HepaRG	Unknown	+[44]	–	+[57]
Huh7	Unknown	+/–	+/–	+[56]
Huh7^NTCP^	Unknown	+[46]	+/–	Unknown
Huh7.5.1	Unknown	–	+[58, 59]	Unknown
HLCZ01	Unknown	+[60]	+[60]	Unknown
PLC/PRF5	Unknown	Unknown	Unknown	+[61]
A549	Unknown	–	–	+[61]
iPSC-derived HLC	Unknown	+[19, 62–64]	+[17, 65]	+[18, 66, 67]
hESC-derived HLC	Unknown	+[19]	+[17, 65]	+[18, 66, 67]

TIM-1: T-cell immunoglobulin and mucin domain 1; NTCP: sodium taurocholate cotransporting polypeptide; CD81: cluster of differentiation 81; SR-BI: scavenger receptor class B type I; OCLN: occluding; CLDN1: claudin-1; PHH: primary human hepatocyte; iPSC: induced pluripotent stem cell; hESC: human embryonic stem cell; HLC: hepatocyte-like cell. +: permissive; +/-: barely permissive; -: not permissive.
